# Electroanatomical mapping–guided left bundle branch area pacing in patients with structural heart disease and advanced conduction abnormalities

**DOI:** 10.1093/europace/euac232

**Published:** 2022-12-30

**Authors:** Sergio Richter, Roman Gebauer, Micaela Ebert, Cathleen Moscoso Ludueña, Dominik Scheller, Johannes Lucas, Sebastian König, Ingo Paetsch, Gerhard Hindricks, Michael Döring

**Affiliations:** Division of Electrophysiology, Heart Center Dresden, Technische Universität Dresden, Fetscherstr. 76, 01307 Dresden, Germany; Department of Electrophysiology, Heart Center, University of Leipzig, Strümpellstr. 39, 04289 Leipzig, Germany; Department of Pediatric Cardiology, Heart Center, University of Leipzig, Strümpellstr. 39, 04289 Leipzig, Germany; Division of Electrophysiology, Heart Center Dresden, Technische Universität Dresden, Fetscherstr. 76, 01307 Dresden, Germany; Department of Electrophysiology, Heart Center, University of Leipzig, Strümpellstr. 39, 04289 Leipzig, Germany; Department of Electrophysiology, Heart Center, University of Leipzig, Strümpellstr. 39, 04289 Leipzig, Germany; Department of Therapy Specialists, Electrophysiology, Abbott Medical GmbH, Helfmann-Park 7, 65760 Eschborn, Germany; Department of Electrophysiology, Heart Center, University of Leipzig, Strümpellstr. 39, 04289 Leipzig, Germany; Department of Electrophysiology, Heart Center, University of Leipzig, Strümpellstr. 39, 04289 Leipzig, Germany; Department of Electrophysiology, Heart Center, University of Leipzig, Strümpellstr. 39, 04289 Leipzig, Germany; Department of Electrophysiology, Heart Center, University of Leipzig, Strümpellstr. 39, 04289 Leipzig, Germany; Department of Electrophysiology, Heart Center, University of Leipzig, Strümpellstr. 39, 04289 Leipzig, Germany

**Keywords:** Left bundle branch area pacing, Electroanatomical mapping, Radiation exposure, Implantation technique, Cardiac implantable electronic device

## Abstract

**Aims:**

Left bundle branch area pacing (LBBAP) can be technically challenging and fluoroscopy-intense. Three-dimensional electroanatomical mapping (EAM) facilitates non-fluoroscopic lead navigation and electrogram mapping. We sought to prospectively evaluate the feasibility, safety, and outcomes of routine EAM-guided LBBAP in patients with structural heart disease (SHD) and advanced conduction abnormalities.

**Methods and results:**

Consecutive patients with SHD and conduction abnormalities who underwent an attempt at EAM-guided LBBAP were included. The feasibility, safety, procedural, and mid-term outcomes were evaluated. Electrical, echocardiographic, and clinical parameters were assessed at implantation and last follow-up. Thirty-two patients (68 ± 18 years; 19% female) were included, of which 75% had intrinsic QRS > 150 ms, 53% left bundle branch block, and 25% right bundle branch block. Primary EAM-guided LBBAP was successful in 29 patients (91%). The procedural duration was 95 (70–110) min, total fluoroscopy time 0.93 (0.40–1.73) min, and total fluoroscopy dose 35.4 (20.5–77.2) cGy cm2. Paced QRS duration (QRSd) was significantly shorter than intrinsic QRSd (121.9 ± 10.7 vs. 159.2 ± 34.4 ms; *P* < 0.001) and remained stable during the mean follow-up of 7.0 ± 5.9 months. The LBBAP capture threshold was 0.57 ± 0.23 V/0.4 ms at implantation and remained low during follow-up (0.58 ± 0.18 V/0.5 ± 0.2 ms; *P* = 0.877). Overall left ventricular ejection fraction improved significantly from 44.2 ± 14.3% at baseline to 49.4 ± 13.1% at follow-up (*P* = 0.009), New York Heart Association class from 2.4 ± 0.6 to 1.8 ± 0.6 (*P* = 0.002), respectively. No complications occurred that required intervention.

**Conclusion:**

Routine near-zero fluoroscopy EAM-guided LBBAP can safely be performed in patients with SHD and advanced conduction abnormalities with high success rates and favourable mid-term outcomes. Further studies are needed to investigate whether the use of EAM improves the overall outcome of conduction system pacing and to identify specific patient populations who benefit the most from EAM-guided lead implantation.

**What’s new?**

- Implementation of routine electroanatomical mapping (EAM)-guided lead implantation into routine clinical practice for left bundle branch area pacing (LBBAP) is feasible and safe in patients with structural heart disease and advanced conduction abnormalities.
- Routine EAM-guided lead implantation enables low-fluoroscopy LBBAP without increasing procedural duration or procedure-related complications.
- Near-zero fluoroscopy EAM-guided LBBAP is associated with high acute success rates and favourable mid-term outcomes.

## Introduction

Conduction system pacing (CSP) achieved by permanent His bundle pacing (HBP) or left bundle branch pacing (LBBP) has been established as an alternative physiological pacing option in patients with an indication for right ventricular pacing (RVP) and cardiac resynchronization therapy (CRT).^1,2^ Direct HBP provides the most physiological form of cardiac pacing and circumvents the potentially deleterious effects of RVP-induced ventricular dyssynchrony.^3^ However, the success rates of HBP are considerably lower in patients with complex structural cardiac abnormalities and infranodal conduction disease, particularly in those with left bundle branch block (LBBB).^4^ In these patients, who likely gain the most benefit from CSP, HBP lead implantation is often technically challenging, fluoroscopy-intense, and requires high precision to map for an appropriate pacing site. The use of three-dimensional (3D) electroanatomical mapping (EAM) to guide HBP lead implantation has been demonstrated to be safe and feasible and is associated with a significant reduction in radiation exposure.^5,6^ Nevertheless, conventional or EAM-guided HBP is limited by its inability to circumvent the site of a more distal LBBB or to correct proximal LBBB with reasonable pacing output. In addition, an unpredictable rise in HBP capture thresholds or loss of His capture is frequently observed during follow-up, which leads to premature battery depletion and an increased need for lead revisions.^7^ Compared with HBP, left bundle branch area pacing (LBBAP) enables physiological pacing with the ability to recruit the Purkinje conduction system more distally and to pace beyond the site of LBBB with stable and low capture thresholds.^8,9^ The recent implementation of LBBAP in clinical practice has increased the feasibility of delivering CSP to most patients irrespective of the underlying conduction disturbance. Although LBBAP requires less precision than HBP, lead implantation can also be challenging and is associated with significant fluoroscopy exposure, particularly in patients with structural heart disease (SHD) and indication for CRT.^2^ Electroanatomical mapping guidance holds great potential to facilitate low-fluoroscopy LBBP lead implantation in these patients, but data on EAM-guided LBBAP are scarce and currently limited to case reports and small case series.^10,11^

The aim of our study was to prospectively evaluate the feasibility, safety, and outcome of routine EAM-guided LBBAP in patients with SHD and advanced conduction abnormalities.

## Methods

### Patient selection

This prospective single-centre observational study included patients with an established indication for antibradycardia pacing or CRT who underwent a primary attempt at routine EAM-guided LBBAP using the Medtronic Select Secure 3830 pacing lead at Heart Center Leipzig. All patients had underlying SHD with or without reduced systolic left ventricular (LV) function and advanced atrioventricular (AV) conduction disease (second- or third-degree AV block and/or complete bundle branch block) with prolonged intrinsic QRS duration (QRSd). Electroanatomical mapping-guided lead implantation was performed in all patients following a standardized protocol irrespective of the pacing indication and type of conduction disturbance. Patients were excluded from analysis if they had undergone LBBAP-optimized CRT, had an upgrade procedure with concomitant transvenous lead extraction or were lost to follow-up. All patients were ≥18 years of age and provided written informed consent. The collection of data was consistent with the policies of the local institutional review board.

### Implantation procedure

#### Device and conventional lead implantation

Pacemaker (PM) and CRT procedures were performed using standard implantation techniques. Depending on the indication for device therapy, active-fixation right atrial (RA) pacing and/or RVP or implantable cardioverter-defibrillator (ICD) leads were implanted in addition to the LBBP lead via the cephalic, axillary, or subclavian vein. Implantation of conventional PM and ICD leads was guided by minimized low-dose fluoroscopy. A 12-lead electrocardiogram (ECG) was continuously recorded at a sweep speed of 100 mm/s and displayed during the entire procedure to monitor changes in paced QRS morphology and differentiate between myocardial, non-selective LBBP (NS-LBBP), and selective LBBP (S-LBBP) capture. Implantation procedures were performed in local anaesthesia and either minimal, conscious, or deep sedation.

#### Electroanatomical mapping-guided lead implantation

We have recently described in detail our refined approach to lead implantation guided by the EnSite Precision system (St Jude Medical, St Paul, MN, USA) for permanent HBP.^6^ Briefly, EAM-guided LBBAP was performed in a similar way using the Select Secure pacing lead (Model 3830, 69 cm; Medtronic, Inc., Minneapolis, MN, USA) delivered through a fixed-curve (C315His) or deflectable (C304His) sheath (Medtronic, Inc., Minneapolis, MN, USA). In order to assure optimal catheter stability and accuracy during EAM, the tip electrode of an implanted bipolar active-fixation RA or right ventricular (RV) lead was used as a reference. The SelectSecure pacing lead was connected to an alligator clamp threshold cable (St. Jude Medical, Minnetonka, MN, USA) in a unipolar fashion and the reference lead to a second alligator clamp threshold cable in a bipolar fashion. The alligator clamp cables were pinned to the joint catheter input module of the electrophysiology (EP) recording system (Prucka Cardiolab, GE Healthcare, Waukesha, WI, USA) and 3D mapping system and connected to the pacing system analyser (Merlin PCS 3650, St Jude Medical, Sylmar, CA, USA) using custom-made jumper cables for simultaneous recording of electrograms and measured electrical data. Following insertion of the delivery sheath into the RA over a long guidewire, the electrically connected SelectSecure pacing lead was advanced non-fluoroscopically just beyond the tip of the sheath to enable unipolar electrogram and pace mapping. The roving lead tip was directly visualized on the map and non-fluoroscopically navigated to the regions of interest with simultaneous acquisition of 3D geometry. Mapping sites with a His bundle (HB) or right bundle branch potential or critical pacing responses were recorded and tagged on the map.

**Figure 1:**
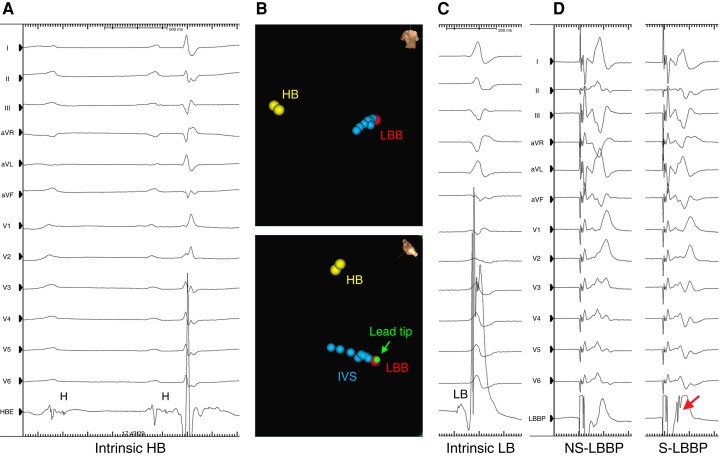
Targeted LBBP guided by the EnSite Precision system in a patient with non-ischaemic CMP and advanced infranodal conduction disease (*A*). Electroanatomical maps with tagged locations at distal HB (yellow dots), IVS (blue dots), and LBB (red dot) in right anterior oblique (upper panel) and left anterior oblique cranial (lower panel) projections; the green icon represents the visualized roving lead tip (green arrow) at the target LBB position (*B*). Twelve-lead ECGs and unipolar electrograms from the lead tip at the mapped HB (*A*) and target LBB position at baseline (*C*) and during NS- and S-LBBP (*D*). Note the LB injury current (*C*) and discrete component on the unipolar electrogram during S-LBBP (*D*, arrow). CMP, cardiomyopathy; ECG, electrocardiogram; HB, His bundle; IVS, interventricular septum; LBBP, left bundle branch (LBB) pacing; NS, non-selective; S, selective.

**Figure 2:**
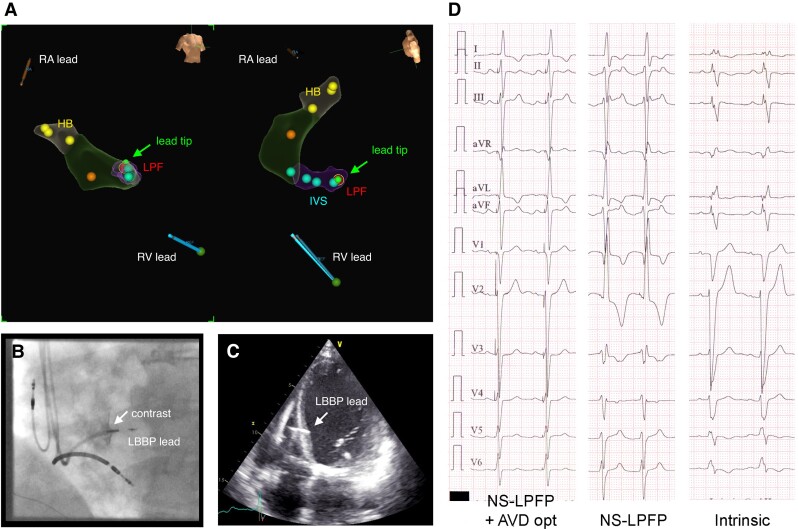
Targeted LBBP guided by the EnSite Precision system in a patient with ischaemic CMP and LBBB. (*A*) Electroanatomical maps with tagged locations at the HB region (yellow dots), IVS (blue dots), and LPF (red dot) in right anterior oblique (left panel) and LAO cranial (right panel) projections; the green icon represents the visualized roving lead tip at the target position. Note the more distal LPFP. (*B*) Contrast fluoroscopic image in LAO projection demonstrating the position of the LBBP lead in the IVS (arrow). (*C*) Echocardiographic image in apical four-chamber view confirming the location of the LBBP lead tip deep in the LV septum (arrow). (*D*) Twelve-lead ECGs at 12-month follow-up demonstrating intrinsic conduction, NS-LPFP only, and NS-LPFP with intrinsic RBB conduction after AVD opt. AVD opt, atrioventricular delay optimization; CMP, cardiomyopathy; ECG, electrocardiogram; HB, His bundle; IVS, interventricular septum; LAO, left anterior oblique; LBBP, left bundle branch pacing; LPFP, left posterior fascicle (LPF) pacing; NS, non-selective; RA, right atrial, RV, right ventricular.

**Figure 3:**
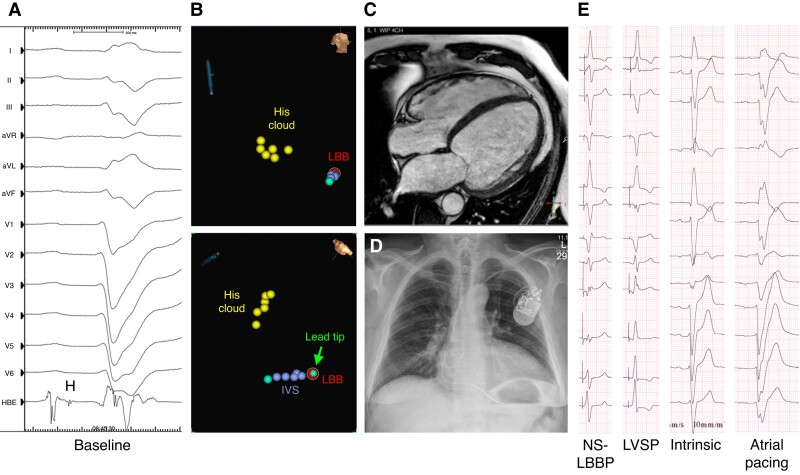
Targeted LBBP guided by the EnSite Precision system in a patient with severe dilated CMP and advanced infranodal conduction disease. (*A*) Twelve-lead ECG and unipolar electrogram from the lead tip at the proximal HB showing LBBB with wide QRS (238 ms) and prolonged HV interval (106 ms). (*B*) Electroanatomical maps with tagged locations at the HB region (yellow dots), right ventricular septum (turquoise dot), IVS (blue dots), and LBB (red dot) in right anterior oblique (upper panel) and left anterior oblique cranial (lower panel) projections; the green icon represents the visualized roving lead tip at the target LBB position. (*C*) Baseline CMR image in apical four-chamber view demonstrating severe dilatation of the left ventricle. (*D*) Post-operative chest radiograph in posterior–anterior view. (*E*) Twelve-lead ECGs at 6-month follow-up demonstrating LBBB and 2:1 conduction in the right bundle during atrial pacing (600 ms), intrinsic conduction with left anterior fascicular block during sinus rhythm (1050 ms); LVSP and NS-LBBP during threshold testing (600 ms). See text for more information. CMP, cardiomyopathy; CMR, cardiovascular magnetic resonance; ECG, electrocardiogram; HB, His bundle; HV, His-ventricular; IVS, interventricular septum; LBBB, left bundle branch block; LBBP, left bundle branch (LBB) pacing; LVSP, left ventricular septal pacing; NS, non-selective.

In all patients we routinely attempted to initially map and annotate the HB in order to (i) determine the His-ventricular (HV) interval; (ii) define the site of conduction block; and (iii) set an electroanatomical landmark for subsequent localization of septal sites suitable for LBBAP. Electroanatomical mapping-guided lead implantation was basically performed following previously described techniques and criteria.^12,13^ Dual electroanatomical maps were simultaneously displayed in right anterior oblique and left anterior oblique cranial projections (*Figures 1–3*). The visualized tip of an apically implanted reference RV lead (if applicable) served as an anatomical landmark (*Figure 2*). The system was then advanced ∼1.5–2 cm anteriorly and inferiorly from the tagged distal HB towards the RV apex, and unipolar pace mapping at an output of 3.0 V/0.4 ms was initiated to identify an appropriate septal site for lead deployment. The septal target site, where the paced QRS morphology usually demonstrated an inferior lead discordance (R or Rs in Lead II, and rS in Lead III) and ‘w-pattern’ in Lead V1, was tagged on the 3D map. Lead deployment was then performed to cross the interventricular septum by means of rapid clockwise rotations. Continuous or intermittent unipolar pacing was conducted during fixation to closely monitor the paced QRS morphology and pacing impedance, and to determine and tag the respective intraseptal depth of the roving lead tip on the 3D map (*Figures 1–3*, blue dots). Lead deployment was halted when the paced QRS morphology revealed a positive terminal deflection (qR, Qr, rSR pattern) in Lead V1 and features of left bundle branch (LBB) capture were evident. All possible efforts were undertaken to obtain LBB capture. Left ventricular septal pacing (LVSP without clear evidence of direct LBB capture) was accepted if further advancement of the lead to the LV subendocardial layer was not feasible or perforation into the LV cavity impended after a number of attempts at different sites. In selected patients, the final lead depth was also confirmed by low-fluoroscopy sheath angiography (*Figure 2B*). Following successful LBBP lead implantation and withdrawal of the guiding sheath into the RA, lead position and slack were confirmed by brief snapshot fluoroscopic image(s). Final slitting of the guiding sheath was performed under minimal low-dose fluoroscopic guidance to assure and document steady lead position and adequate slack. The LBBP lead remained connected to the EAM system throughout these steps to closely monitor lead stability.

### Definitions

Left bundle branch area pacing was defined as successful if the LBBP lead could be permanently implanted deep in the LV septum or at the LBB with a paced QRS morphology demonstrating a positive terminal deflection (qR, Qr, rSR pattern) in Lead V1 along with an acceptable capture threshold (<1.5 V/0.4 ms) and ventricular sensing (>5 mV). Determination of LBB capture was based on previously published criteria^13^ and included in addition to right bundle branch block (RBBB) configuration with a positive terminal deflection in Lead V1 during unipolar tip pacing ≥1 of the following criteria: (i) recording of an LBB potential in non-LBBB patients with equal intrinsic and paced R wave peak time (RWPT) in Lead V6 (±10 ms) (*Figure 1*); (ii) demonstration of a diagnostic QRS morphology transition from NS-LBBP to S-LBBP (constant RWPT) or septal myocardial capture (abrupt increase in RWPT >10 ms) during threshold testing or programmed deep septal stimulation; (iii) V6–V1 interpeak interval >40 ms. If direct LBB capture could not be confirmed, LVSP was considered to be present. RWPT was measured from stimulus to the peak of the R wave in Lead V_6_. Intrinsic and paced QRSd was measured from the earliest QRS onset to the latest QRS offset in any of the 12 surface leads. Fluoroscopy times and doses were recorded for the overall procedure and separately for LBBP lead implantation. Left bundle branch pacing lead timing included guiding sheath introduction and removal.

### Follow-up

Follow-up was performed predischarge, at 4 weeks, 2 months, and then every 3–6 months post-implantation. At each outpatient visit, the clinical status and device function were assessed. Left bundle branch area pacing threshold testing including assessment of the type of LBB capture was performed during continuous 12-lead ECG recording at a sweep speed of 25 mm/s. Lead-related complications and significant changes in pacing parameters were routinely tracked. Devices were programmed based on the indication, complexity of the system, and patients’ individual needs. Patients scheduled for the pace-and-ablate strategy underwent AV nodal ablation (AVNA) usually at a 4-week follow-up after confirmation of good and stable lead parameters. Echocardiographic follow-up was usually performed at the earliest 8–12 weeks post-implantation. The echocardiographic response was defined as ≥5% increase in LV ejection fraction (LVEF). Data from the last follow-up visit were used for analysis. A minimum device follow-up of 4 weeks was required for the purpose of the study.

### Statistical analysis

Data are expressed as numbers and percentages for categorical variables and mean ± standard deviation (SD) or median with interquartile ranges (IQRs) for continuous variables. Descriptive statistics were reported for the overall study cohort. The McNemar test was used to compare categorical variables between paired groups. Continuous variables between groups were analysed with the paired *t*-test or Wilcoxon signed-rank test, as appropriate. Tests were two-tailed, a probability value <0.05 was considered statistically significant. All statistical analyses were performed using the SPSS software (Version 26.0, IBM Corp., Armonk, NY, USA).

## Results

### Baseline characteristics

The study cohort consisted of 32 patients (mean age 68 ± 18 years; 19% female) who underwent a primary attempt at EAM-guided LBBAP. The baseline characteristics of the study population are summarized in *Table 1*. All patients had SHD and concomitant conduction abnormalities. The most frequent underlying disorder was coronary artery disease in 13 patients (41%), 7 patients (22%) had a previous myocardial infarction and 9 patients (28%) resultant ischaemic cardiomyopathy (CMP). Non-ischaemic CMP was present in 11 patients (34%). Thirty-four per cent of patients had previous surgical or interventional aortic (*n* = 10) or mitral (*n* = 1) valve replacement. The mean baseline QRSd of the study cohort was 160 ± 34 ms. Twenty-four patients (75%) had an intrinsic QRSd >150 ms, 17 patients (53%) presented with LBBB and 8 patients (25%) with RBBB. Among those with RBBB, five patients had bifascicular block. The mean baseline LVEF of the study cohort was 45 ± 14%. Twenty patients (62%) presented with reduced LV systolic function (LVEF < 50%), of which 10 patients had LVEF ≤ 35% (31% of the entire cohort). The leading indication for LBBAP was CRT in 50%, intermittent or persistent AV block in 44%, and refractory atrial fibrillation prior to AVNA (pace-and-ablate) in 6% of the cases. Fifteen patients (47%) of the entire cohort received a dual-chamber PM, 14 patients (44%) a biventricular device, and 3 patients (9%) a single-chamber PM. Patients were followed for a mean duration of 7.0 ± 5.9 [median 5.4 (IQR: 2.1–12.6)] months.

**Table 1:** Baseline characteristics of the study population

	All patients (*n* = 32)
Age, years	68 ± 18
Male, *n* (%)	26 (81)
Hypertension, *n* (%)	25 (78)
Diabetes mellitus, *n* (%)	9 (28)
Atrial fibrillation, *n* (%)	15 (47)
Underlying structural heart diseases^a^	
ȃHypertensive heart disease	8 (25)
ȃCoronary artery disease, *n* (%)	13 (41)
ȃȃPrevious myocardial infarction	7 (22)
ȃIschaemic CMP, *n* (%)	9 (28)
ȃNon-ischaemic CMP, *n* (%)	11 (34)
ȃPost-aortic or mitral valve intervention, *n* (%)	11 (34)
ȃȃTAVI, *n* (%)	5 (16)
ȃȃSurgical AVR, *n* (%)	5 (16)
ȃȃSurgical MVR, *n* (%)	1 (3)
ȃOther^b^	2 (6)
Baseline electrocardiographic parameters	
ȃSinus rhythm, *n* (%)	24 (75)
ȃPR interval, ms	261 ± 92
ȃQRS duration, ms	160 ± 34
ȃȃQRS duration >150 ms, *n* (%)	24 (75)
ȃLBBB, *n* (%)	17 (53)
ȃRBBB, *n* (%)	8 (25)
ȃȃRBBB + LFB, *n* (%)	5 (16)
ȃNon-BBB, *n* (%)	7 (22)
Baseline echocardiographic parameters	
ȃLVEF, %	45.0 ± 14.2
ȃȃLVEF 36–50%, *n* (%)	10 (31)
ȃȃLVEF ≤35%, *n* (%)	10 (31)
ȃLVEDV, mL	165.0 ± 92.0
ȃLVEDD, mm	54.1 ± 8.3
ȃIVS, mm	12.1 ± 2.7
Indications for pacing	
ȃAVB, *n* (%)	14 (44)
ȃCRT, *n* (%)	16 (50)
ȃȃUpgrade to CRT, *n* (%)	7 (22)
ȃPace-and-ablate, *n* (%)	2 (6)
Type of implanted device	
ȃSingle-chamber PM, *n* (%)	3 (9)
ȃDual-chamber PM, *n* (%)	15 (47)
ȃBiventricular PM, *n* (%)	5 (16)
ȃBiventricular ICD, *n* (%)	9 (28)

Data are presented as mean ± SD or number (percentage).

AVB, atrioventricular block; AVR, (transcatheter or surgical) aortic valve replacement; CMP, cardiomyopathy; CRT, cardiac resynchronization therapy, ICD, implantable cardioverter-defibrillator; IVS, interventricular septum; LBBB, left bundle branch block; LFB, left fascicular block; LVEDD, left ventricular end-diastolic diameter; LVEDV, left ventricular end-diastolic volume; LVEF, left ventricular ejection fraction; MVR, mitral valve replacement or repair; PM, pacemaker; RBBB, right bundle branch block; TAVI, transcatheter aortic valve implantation.

Several patients presented with >1 finding.

Including one patient with foetal alcohol syndrome after surgical ventricular septal defect repair; one patient with hypertrophic CMP (IVS = 18 mm).

### Procedural outcomes

Primary EAM-guided LBBAP was successful in 29 patients (91%). Two patients with a failed attempt to LBBAP were switched to conventional biventricular pacing. The other patient finally underwent successful EAM-guided HBP. Reasons for failure were the inability to advance the lead to a deep septal position (no terminal *r*/*R*) in two patients and the inability to recruit the LBB at reasonable pacing output (<3.0 V/1.0 ms) in one patient with LBBB. Hence, the overall success rate for EAM-guided CSP (LBBAP and HBP) was 94%. Two minor procedure-related complications occurred, one pocket haematoma on intravenous heparin and one small pneumothorax. Both complications could be managed conservatively. Procedural outcomes are summarized in *Table 2*.

**Table 2:** Procedural outcome

Implant success	Total attempts to EAM-guided LBBAP (*n* = 32)
Successful EAM-guided LBBAP, *n* (%)	29/32 (91)
Switch to EAM-guided HBP, *n* (%)	1/32 (3)
Switch to conventional BiVP, *n* (%)	2/32 (6)
**Procedural characteristics**	**Successful EAM-guided cases (*n* = 30)**
Procedural time, min	95 (70–110)
Total fluoroscopy time, min	0.93 (0.40–1.73)
Total fluoroscopy dose, cGy cm2	35.5 (20.5–77.2)
Fluoroscopy time—first HBE recording, min	0.01 (0.00–0.01)
Fluoroscopy dose—first HBE recording, cGy cm^2^	0.30 (0.00–0.85)
LBBP lead fluoroscopy time, min	0.82 (0.23–1.33)
LBBP lead fluoroscopy dose, cGy cm^2^	28.4 (15.5–58.9)
Electrogram and LBBP lead parameters	
Intrinsic QRS duration	159.2 ± 34.4
HBE recording, *n* (%)	29 (97)
HV interval, ms	64.0 (54.5–74.0)
ȃHV interval >70 ms, *n* (%)	8/28 (29)
LBB potential recording, *n* (%)	13 (43)
LBB potential to QRS onset, ms	25.1 ± 8.7
Paced QRS duration, ms	121.9 ± 10.7
LBB capture, *n* (%)	24/29 (83)
ȃNS-to-S-LBBP transition	7/29 (24)
ȃNS-LBBP to LVSP transition	17/29 (59)
LVSP only, *n* (%)	5/29 (17)
RWPT, ms	79.7 ± 13.1
Pacing threshold, V/0.4 ms	0.57 ± 0.23
Pacing impedance, Ω	654 ± 114
Ventricular sensing, mV	8.9 ± 2.7

Data are presented as median (IQR), mean ± SD, or number (percentage).

EAM, electroanatomical mapping; BiVP, biventricular pacing; HBE, HB electrogram; HBP, His bundle (HB) pacing; HV, His-ventricular; LBB, left bundle branch; LBB(A)P, left bundle branch (area) pacing; LVSP, left ventricular septal pacing; NS, non-selective; RWPT, R wave peak time in lead V_6_; S, selective.

#### Procedure time and fluoroscopy exposure

**Figure 4:**
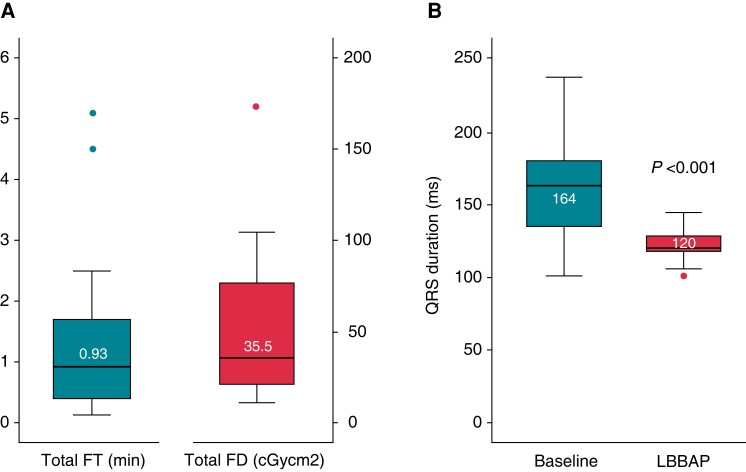
Procedural outcomes of EAM-guided LBBAP. Median total fluoroscopy time (FT) and fluoroscopy dose (FD) (*A*) and changes in QRSd with LBBAP (*B*) in the overall population. EAM, electroanatomical mapping; LBBAP, left bundle branch area pacing.

The median total procedural duration was 95 (70–110) min. The median total fluoroscopy time and dose were 0.93 (0.40–1.73) min and 35.4 (20.5–77.2) cGy cm2, respectively (*Figure 4A*). The median LBBP lead fluoroscopy time was 0.82 (0.23–1.33) min, the LBBP lead fluoroscopy dose 28.4 (15.5–58.9) cGy cm2. Mapping and recording of the first HB electrogram were achieved virtually non-fluoroscopically in all patients with preserved AV nodal conduction [median fluoroscopy time 0.6 (0.0–0.6) s, fluoroscopy dose 0.3 (0.0–0.9) cGy cm2].

#### Electrogram and left bundle branch pacing lead parameters

An HB potential was initially mapped and recorded in all but one patient with a complete nodal AV block (97%). The median HV interval was 64 (55–74) ms. Eight patients (29%) had an HV interval >70 ms, four patients (13%) had second- (*n* = 3) or third-degree (*n* = 1) infrahisian conduction block. An LBB potential was recorded in 13 patients (43%) with a mean interval from LBB potential to QRS onset of 25.1 ± 8.7 ms. The mean paced QRSd was significantly shorter compared with the baseline intrinsic QRSd (121.9 ± 10.7 vs. 159.2 ± 34.4 ms; *P* < 0.001) (*Figure 4B*) and remained stable during follow-up (120.3 ± 11.1 ms; *P* = 0.370). The mean RWPT during LBBAP at implantation was 79.7 ± 13.1 ms. Left bundle branch capture was present in 83% of patients. A transition from NS-LBBP to S-LBBP was demonstrated in 24% and a transition from NS-LBBP to LVSP in 59% of the cases. Two patients with transition from NS-LBBP to LVSP at implantation experienced loss of direct LBB capture during follow-up. The LVSP capture threshold and ventricular sensing amplitude remained stable in both patients. The mean LBBAP capture threshold at implantation was 0.57 ± 0.23 V at 0.4 ms and remained low during follow-up (0.58 ± 0.18 V at 0.5 ± 0.2 ms; *P* = 0.877). The ventricular sensing amplitude at implantation was 8.9 ± 2.7 mV and significantly increased to 10.4 ± 2.5 mV during follow-up (*P* = 0.004) (*Table 3*). The patient who underwent permanent HBP had a stable His capture threshold (0.5 V/0.4 ms) and ventricular sensing (3.8 mV) at the last follow-up.

**Table 3:** Electrical, echocardiographic, and clinical outcomes

	Implantation	Last follow-up	*P*-value
Successful EAM-guided cases, *n* (%)	30 (94)		
Time to last follow-up, months	5.4 (2.1–12.6)		
Electrical parameters			
Pacing threshold, V @ ms	0.57 ± 0.23 @ 0.4	0.58 ± 0.18 @ 0.5 ± 0.2	0.877
Pacing Impedance	654 ± 114	468 ± 93	<0.001
Ventricular sensing, mV	8.9 ± 2.7	10.4 ± 2.5	0.004
Paced QRS duration, ms	121.9 ± 10.7	120.3 ± 11.1	0.370
LBB capture, *n* (%)	24/29 (83)	22/29 (76)	0.500
HB capture, *n* (%)	1/1 (100)	1/1 (100)	1.000
Echocardiographic parameters			
LVEF, %	46.5 (31.2–56.5)	53.0 (45.0–60.0)	0.009
Baseline LVEF ≤35%, %	28.5 (25.0–32.8)	37.5 (19.5–48.3)	0.124
LVEDV, mL	182.2 ± 107.3	162.8 ± 75.9	0.128
LVEDD, mm	55.5 ± 9.3	53.1 ± 8.4	0.053
Clinical parameters			
NYHA class	2.4 ± 0.6	1.8 ± 0.6	0.002

Data are presented as median (IQR), mean ± SD, or number (percentage).

EAM, electroanatomical mapping; HB, His bundle; LBB, left bundle branch; LBBAP, left bundle branch (LBB) area pacing; LVEDD, LV end-diastolic diameter; LVEDV, LV end-diastolic volume; LVEF, left ventricular ejection fraction; LVSP, left ventricular septal pacing; NYHA, New York Heart Association.

### Clinical and echocardiographic outcomes

No device/lead-related complications occurred during a mean follow-up duration of 7.0 ± 5.9 months [median 5.4 (IQR: 2.1–12.6) months], and no patient died. None of the study patients experienced lead perforation or dislodgement and there was no increase in LBBAP capture threshold >1.0 V/0.4 ms observed during follow-up. All patients had good and stable electrical parameters at the last follow-up. Electrical, echocardiographic, and clinical parameters at implantation and last follow-up are listed in *Table 3*.

**Figure 5:**
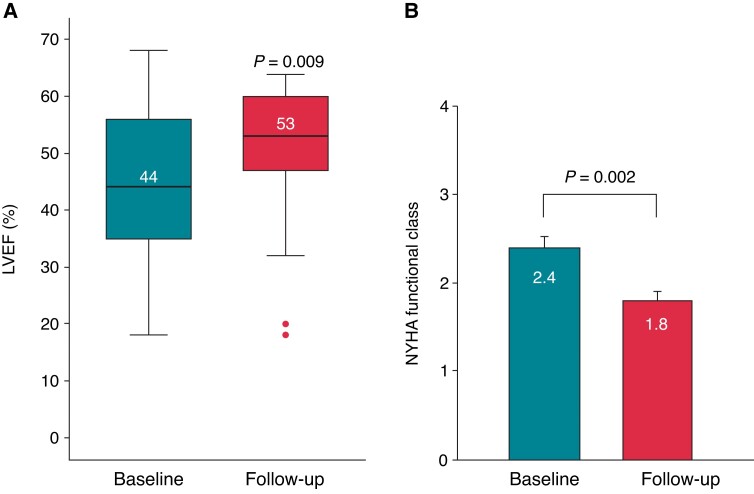
Echocardiographic (*A*) and clinical (*B*) responses of the entire cohort after left bundle branch area pacing. LVEF, left ventricular ejection fraction; NYHA, New York Heart Association.

Because of the COVID-19 pandemic, echocardiographic follow-up data were only available for 21 of 30 successfully implanted patients (70%) and obtained after a mean time of 7.1 ± 5.9 [median 4.8 (IQR: 2.2–11.6)] months post-implantation. Overall LVEF improved significantly from 44.2 ± 14.3% at baseline to 49.4 ± 13.1% at the last follow-up (*P* = 0.009). Echocardiographic response (ΔLVEF > 5%) was observed in 48% of the examined patients. Among patients with baseline LVEF ≤ 35%, LVEF increased from 28.4 ± 6.1% to 34.9 ± 12.6% without reaching statistical significance (*P* = 0.124) (*Figure 5A*). There were no significant changes in LV end-diastolic diameter (LVEDD; 55.5 ± 9.3 vs. 53.1 ± 8.4 mm; *P* = 0.053) or LV end-diastolic volume (LVEDV; 182.2 ± 107.3 vs. 161.7 ± 77.0 mL; *P* = 0.128). Clinical response, defined as improvement in ≥1 New York Heart Association (NYHA) functional class, was noted in 58% of patients. There was a significant improvement in the overall NYHA functional class from 2.4 ± 0.6 at baseline to 1.8 ± 0.6 at the last follow-up (*P* = 0.002) (*Figure 5B*).

Two patients with baseline LVEF ≤35% and no improvement in LVEF and NYHA functional class presented with atrial fibrillation with a fast ventricular response and a low percentage of LBBAP at the time of the last echocardiographic follow-up. Another patient with severe dilated CMP and LBBB, in whom LVEF did not significantly increase with NS-LBBP (LVEF 18→20%), reported on clinical response (NYHA III/IV→II) at 6-month follow-up. Cardiovascular magnetic resonance (CMR) revealed reverse structural remodelling with decreases in LV dimensions (LVEDD 81→74 mm) and volumina (LVEDV 571→ 413 mL), which further resulted in reverse electrical remodelling with narrowing of the intrinsic QRSd and improvement from complete LBBB (QRSd 238 ms) to left anterior fascicular block (QRSd 185 ms) at normal heart rates (*Figure 3*).

## Discussion

The present study prospectively evaluated the feasibility and safety of routine EAM-guided LBBAP in patients with moderate to severe SHD and advanced conduction abnormalities. To the best of our knowledge, this is the largest EAM-guided LBBAP series published to date. The primary findings of our study are as follows: (i) routine EAM-guided LBBAP is feasible and safe in this subset of patients with an acute implantation success rate of 91%; (ii) EAM-guided LBBP lead implantation is associated with very low fluoroscopy exposure and no relevant increase in the procedural duration or procedure-related complications as compared with published data on conventional lead implantation; and (iii) electrical lead parameters remained stable and LBBAP resulted in significant clinical and echocardiographic improvements during mid-term follow-up.

Conventional fluoroscopy-guided CSP lead implantation can be technically challenging, time-consuming, and fluoroscopy-intense in patients with complex structural abnormalities and infranodal conduction disease. Three-dimensional EAM has recently been implemented into HBP procedures to facilitate non-fluoroscopic visualization and navigation of the HBP lead and mapping of the HB region.^5,6^ In a previous study on routine EAM-guided HBP, we demonstrated that HBP lead implantation guided by 3D EAM was feasible and particularly helpful in patients with infranodal conduction disease and resulted in a tremendous reduction in fluoroscopy exposure compared with conventional lead implantation.^6^ Even though 3D EAM allows for detailed high-density electrogram and pace mapping of the distal HB area, correction of infranodal AV block or LBBB may not be possible or requires high capture thresholds in a significant number of patients (∼20–40%).^4^ The main reason for failure to correct infranodal conduction block is basically that the site of the block is located either beyond the distal HB or within the distal HB, but which early penetrates the membraneous septum and is encapsulated by electrically inert fibrous tissue preventing corrective HBP at the reasonable output.^14,15^ With the advent of LBBAP, CSP distal to the HB has become feasible in most patients with stable and low capture thresholds. The target zone for LBBAP is much wider and located within the deep muscular septum, which may explain the high success rates and low capture thresholds that were observed in our and other studies.^16,17^ Moreover, the ventricular sensing amplitudes were higher as usually noted with HBP and significantly increased during follow-up.

Similar to the results of our previous study on HBP, EAM-guided LBBAP was associated with a very low median total fluoroscopy time [0.93 (IQR: 0.40–1.73) min], which was considerably lower than that reported in the literature for conventional LBBAP in patients with and without infranodal conduction disease.^2,16,17^ In two large studies published on LBBAP including 321 and 305 successfully implanted patients, the mean total fluoroscopy times were 13.0 ± 6.7 and 10.4 ± 8.1 min, respectively.^16,17^ Interestingly, the fluoroscopy doses were not reported in either study, although the fluoroscopy dose and not the time is key to the reduction of overall radiation exposure and cancer risk of patients and catheterization laboratory staff.^18^ In our study, the low total fluoroscopy time was accompanied by a very low median dose area product of 35.5 (IQR: 20.5–77.2) cGy cm2 despite triple-lead implantation in 40% of the cases. In this context, it is noteworthy to mention that the final lead depth was estimated on the 3D map and not routinely by contrast fluoroscopy in most patients. In addition, lead stability during withdrawal and slitting of the guiding sheath was usually monitored on the 3D map and required only minimal fluoroscopic guidance.

The feasibility and usefulness of 3D EAM to guide LBBP lead implantation have initially been demonstrated by Vijayaraman *et al*.^10^ in a small series of three cases. However, the mean fluoroscopy time was 10.0 ± 2.6 min despite the use of non-fluoroscopic navigation. Likewise, the median fluoroscopy time was 24.0 min in a small series of five patients with complex anatomy who underwent EAM-guided CSP (four HBP and one LBBAP).^11^ These findings may likely be attributed to the complexity of the cases and EAM protocol used, which included detailed mapping of the HB, RA, and RV with multielectrode catheters. In contrast, we have refined and implemented a standardized protocol for EAM-guided CSP with no need for initial acquisition of 3D right heart geometry, as previously described.^6^ Targeted EAM of the HB and RV septal locations of interest (*Figures 1–3*) has thus been performed without the use of navigation-enabled EP catheters or the need for femoral access, which also limited additional financial costs. Similar to the conventional approach, non-fluoroscopic EAM-guided unipolar mapping was immediately started as soon as the LBBP lead had been advanced to the tip of the guiding sheath. Hence, no additional procedure time was required for mapping. The median total procedure time was 95 (IQR 70–110) min in the present study, which is well in line with that reported for conventional LBBAP.^2,8,16^

Three-dimensional EAM guidance was particularly helpful in cases with challenging anatomy or severe SHD. The possibility to annotate any lead tip position and mapping point of interest on the 3D map enabled not only to easily navigate back and forth to any tagged location with high precision and without fluoroscopy but also to visualize lead progress within the septum and monitor lead depth in real time during LBBP lead deployment (*Figures 1–3*, blue dots). Other useful features of 3D EAM include voltage mapping, which may be of particular interest in patients with ventricular septal defect repair; image integration of CMR-derived 3D anatomical models in patients with complex anatomy or congenital heart disease;^19^ and QRS pattern matching to compare pacing responses and identify the optimal implantation site. Delineation of the cardiac anatomy and conduction system course may also be beneficial in selected cases such as patients with transposition of the great arteries.^20^

### Study limitations

This is a prospective single-centre observational study. Hence, the major limitations are imposed by the non-randomized nature and narrow sample size of the present study. Comparison with a control group of conventionally implanted patients was not performed since selection bias existed in the choice to use EAM preferentially in patients with SHD and advanced conduction abnormalities. Echocardiographic follow-up could not be performed in all patients because of the COVID-19 pandemic, which may have resulted in an underestimation of the echocardiographic response to EAM-guided LBBAP. Moreover, the long-term safety of permanent LBBAP with regard to electrical parameters, lead integrity, and potential lead extraction is still not well established and needs further investigation.

## Conclusions

Routine EAM-guided LBBAP was feasible and safe in patients with SHD and advanced conduction abnormalities and resulted in very low radiation exposure without increasing procedural duration or procedure-related complications. This study confirms that near-zero fluoroscopy lead implantation is associated with high acute success rates and favourable mid-term outcomes. However, further studies are needed to investigate whether the use of EAM improves the overall outcome of CSP and to identify specific patient populations who benefit the most from EAM-guided lead implantation.

## Data Availability

The data underlying this article will be shared on reasonable request to the corresponding author.
